# Modulation of Lipoprotein Cholesterol Levels in *Plasmodium berghei* Malarial Infection by Crude Aqueous Extract of *Ganoderma lucidum*

**DOI:** 10.1155/2012/536396

**Published:** 2012-07-25

**Authors:** Olarewaju M. Oluba, Augustine O. Olusola, George O. Eidangbe, Leye J. Babatola, E. Chukwu Onyeneke

**Affiliations:** ^1^Department of Biochemistry, College of Natural Science, Joseph Ayo Babalola University, Ikeji-Arakeji, Osun, Nigeria; ^2^Department of Biochemistry, Faculty of Science, Adekunle Ajasin University, Akungba Akoko, Ondo, Nigeria; ^3^Department of Medical Biochemistry, College of Medicine, Ambrose Alli University, Ekpoma, Nigeria; ^4^Department of Biochemistry, Faculty of Life Sciences, University of Benin, Benin, Nigeria

## Abstract

In this study, attempt is made to establish changes in serum and liver lipoprotein cholesterols accompanying *Plasmodium berghei* malarial infection in mice treated with aqueous extract of *Ganoderma lucidum* at 100, 250, and 500 mg/kg body weight in comparison with 15 mg/kg chloroquine (CQ). Significant increases in all the lipoprotein fractions were observed in infected untreated mice compared with normal control mice. Treatment with 100 and 250 mg/kg *G. lucidum* extract produced significant reduction in serum total cholesterol (TC) and low-density cholesterol (LDL-C) contents compared with 500 mg/kg *G. lucidum* and CQ. Treatment with CQ, however, produced significant reduction in hepatic TC and LDL-C compared with the extract. A dose-dependent significant increase in serum high-density lipoprotein cholesterol (HDL-C) was observed in the *G. lucidum* treated mice compared with normal control but significantly lower compared with CQ-treated mice. Liver HDL-C level was significantly higher in CQ-treated mice compared with normal control and significantly lower compared with *G. lucidum*-treated and infected untreated mice. A dose-dependent effect of the extract was observed in both serum and liver very-low density lipoprotein cholesterol (VLDL-C). The implication of these results is discussed with respect to the parasite survival and proliferation in the serum and liver.

## 1. Introduction

 The malarial parasite has been observed to have a tremendous requirement for lipids during the replicative stages that take place in the mammalian host. Biochemical studies on blood-stage *Plasmodium* malarial parasites have demonstrated the parasite's proficiency at scavenging and modifying lipids obtained from the host [1]. Studies have also shown that the parasites could obtain free fatty acids (FFAs) directly from the serum or from sources such as high-density lipoprotein (HDL) [2–4]. Within the parasite, scavenged phospholipids can be incorporated without modification [3, 5]. In addition, *Plasmodium* parasites have the capacity to modify fatty acids as needed by elongation or desaturation and incorporate them into phospholipids, diacylglycerols (DAGs), and triacylglycerols (TAGs) [6, 7].

 Indeed, such biochemical revelations have not been possible for liver-stage parasites because of the difficulty in isolating the parasite from the host hepatocytes and the low rate of infection during this stage. However, early studies of malaria in genetically obese Zucker rats (fa/fa) gave indication that host cell lipids are important for *Plasmodium* liver-stage development [8]. These studies demonstrated that obese Zucker rats, which have hepatocytes with increased fatty acid synthesis, triglyceridemia and triglyceride content, supported growth of four times the number of liver-stage schizonts than their lean counterparts. Additionally, 45 hours after infection, schizonts in the obese rats were observed to be twice the size of those in control rats.

 While most scavenged and synthesized lipids are likely incorporated into the membranes of the rapidly growing parasite, additional roles for fatty acids remain to be elucidated. For example, studies have yet to identify the source of fatty acids that are incorporated into GPI, which anchors numerous proteins, including MSP-1, to the merozoite plasma membrane. The low expression of MSP-1 in *fab* knockout parasites suggests that the FAS-II pathway may act together with elongases and desaturases to generate the lipid anchor for some surface proteins, including merozoite surface protein-1 (MSP-1) [9]. Alternatively, the fatty acid synthesis type II (FAS-II) pathway activity may provide a signal for progression to the merozoite formation phase of intrahepatic development. The specific upregulation of the FAS-II pathway during the liver stage may allow the parasite to synthesize its own lipids to supplement those provided by the host hepatocytes during a particularly explosive period of growth in the *Plasmodium* life cycle.

 Elucidating stage-specific requirements for import, synthesis, and utilization of fatty acids and lipids in malarial parasite infection is both of considerable promise for developing novel antimalarial intervention strategies and of fundamental interest in understanding how these parasites are so successful in establishing infection in the human host. Thus, monitoring of serum and liver lipid concentrations during malarial infection could be of immense contribution in establishing the parasite source of lipids for its propagation.

 Mushrooms have been known to have medicinal properties since 1200AD in Chinese folk medicine [10]. In recent years there has been renewed interest in cholesterol lowering properties of mushrooms, including *Ganoderma lucidum* (Reishi mushroom) [11, 12], *Pleurotus ostreatus* (Oyster mushroom) [13], and *Agaricus bisporus* (champignon) [14]. In this study, our focus is *G. lucidum*, an important medicinal fungus belonging to the Ganodermataceae family that has been studied for its many interesting health promoting properties, including antitumour, anti-inflammatory, antiplatelet aggregation, antidiabetic, and hypocholesterolemic properties [15–19]. The cholesterol lowering property of *G. lucidum* is explored in this study for its possible effects in preventing the proliferation of malarial parasites in mice.

## 2. Materials and Methods

### 2.1. Fungal Materials

Fruiting bodies of *G. lucidum* were obtained locally from open forest at Ipele in Ose Local Government Area of Ondo State, Nigeria. The fungal material was identified by Dr. S. Fakoya of the Department of Biological Sciences, Joseph Ayo Babalola University, Ikeji-Arakeji, Osun State, Nigeria, where voucher specimen number 1103 has been deposited.

### 2.2. Extract Preparation

Fruiting bodies of *G. lucidum* were sorted, washed to remove debris and dust particles, and then dried under shade for two weeks. The dried materials were cut into pieces and ground into powder using mechanical grinder. The powdered sample was kept in airtight container and stored at 4°C until required for further analysis. One hundred gram (100 g) of the powdered fungal material was soaked in 1000 mL distilled water and placed in an orbital shaker at room temperature for 48 hr. The aqueous extract was filtered with a muslin cloth. The resulting filtrate was evaporated to dryness at 37°C by the help of a Speed Vac (Model 7811001, Labconco, USA). The recovered extract was weighed and formulated in distilled water to give the required dose.

### 2.3. Animals and Treatment

A total of forty-eight (48) male Swiss albino mice (15–22 g body weight) obtained from the animal laboratory of the Department of Medical Biochemistry, University of Ibadan, Nigeria were used for the study. The animals were housed in stainless steel cages with raised wired floor at 30°C under standard conditions of humidity and 12 h light/dark cycle. They were fed standard feed (Guinea Feeds Ltd) and water *ad libitum*. The animals were kept for an initial period of two feed to acclimatize with their new environment. Before the commencement of the experiment animals were fasted overnight (but allowed access to water *ad libitum*) and weighed. All experimental protocols complied with NIH guidelines [20].

### 2.4. Malarial Parasite

Chloroquine sensitive *Plasmodium berghei *berghei (NK-65) obtained from the Institute for Advanced Medical Research and Training (IMRAT), University College Hospital, University of Ibadan, Nigeria, and kept at the Department of Biochemistry, Joseph Ayo Babalola University, Ikeji-Arakeji, was used for the study. The parasite was subsequently maintained in our laboratory by serial blood passage from mouse to mouse [21] every four days.

### 2.5. Inoculums

One milliliter parasitized erythrocytes were obtained from a donor-infected mouse by cardiac puncture in heparin and made up to 5 mL with normal saline. Mice were inoculated intraperitoneally with 0.2 mL blood suspension containing 10^6^–10^7^ parasitized erythrocytes on day zero. Infected mice with parasitemia of 10–12% were allocated to five groups. The fungal extract and the standard drug (CQ) administration were carried out for four days postinfection.

### 2.6. Treatment Regimen

Forty-eight mice (consisting of 40 infected divided into five groups of eight animals each; a sixth group of eight normal mice, as positive control) were used for this study. The four day postinfection treatment commenced at the establishment of parasitemia in the mice. The treatment administered to each group is as follows:

- negative control—infected mice treated with 0.2 mL normal saline;
- extract test group I (AQ_100_)—infected mice treated with 100 mg/kg *G. lucidum* aqueous extract;
- extract test group II (AQ_250_)—infected mice treated with 250 mg/kg *G. lucidum *aqueous extract;
- extract test group III (AQ_500_)—infected mice treated with 500 mg/kg *G. lucidum *aqueous extract;
- reference test group (CQ)—Infected mice treated with 15 mg/kg chloroquine phosphate;
- positive control—normal mice treated with 0.2 mL distilled water.

Treatments were given once daily by gavage using intubator for four consecutive days according to the method of Builders et al. [22]. On the fifth day (postinfection) thick smears of blood films were obtained from the peripheral blood on the tail from each mouse and used in the determination of the suppressive effect of the extract on the parasitemia level. Thereafter, extract and drug treatment were stopped while mice in each group were placed under surveillance until signs of death were noticed in the infected untreated group (negative control) after which mice in each group were sacrificed.

### 2.7. Blood Collection and Serum Preparation

As soon as signs of death were noticed in the infected untreated mice group (negative control) mice in each group were weighed anesthetized (in chloroform saturated chamber) and sacrificed by jugular puncture. Blood was obtained through their jugular vein into plain sterile bottles and allowed to clot for 2 h and then centrifuged at 3,000 ×g for ten minutes at room temperature to obtain the sera. The sera samples were collected by aspiration using a pasture pipette into sterile bijou bottles and stored frozen until required for analysis, which was done within 72 h.

### 2.8. Tissue Preparation

Upon sacrifice, liver and kidneys of each mouse were quickly excised, rinsed in normal saline, blotted dry on tissue paper, and weighed. Each tissue was placed in separate plastic vial containing ice-cold normal saline and stored at −8°C until required for further analysis.

### 2.9. Preparation of Tissue Homogenate

A weighed portion of liver and kidneys of each mouse was cut out, chopped into small pieces and homogenized using precooled pestle and mortar in a bowl of ice cubes. A 5% homogenate of each tissue was obtained in buffer solution (50 mM Tris-HCl) using normal saline solution, and stored at −8°C until further analysis was carried out.

### 2.10. Assays

Total cholesterol (TC), low-density lipoprotein (LDL) cholesterol, and high-density lipoprotein (HDL) cholesterol were determined using commercial kits (Randox Laboratory Ltd., UK) following the manufacturer's instruction while very low-density lipoprotein (VLDL) cholesterol concentration was determined by difference.

### 2.11. Statistical Analysis

Results presented are means ± SEM of eight independent determinations. Results obtained from this study were statistically analyzed using one way analysis of variance (ANOVA) and Duncan's Multiple Range Test (DMRT) using SPSS 17.0. Significant differences between the treatment means were determined at 95% confidence level.

## 3. Results

 The effects of the extracts treatment compared with CQ and SP as reference drugs on percentage suppression and mean survival time are presented in Table 1. There was no significant difference in % suppression observed in mice treated with 100 mg/kg and those treated with 250 mg/kg aqueous extract and aqueous 200 and aqueous 500 groups, though a significant decrease was noticed in % suppression on comparing aqueous 100 and aqueous 500 treated groups.

 From Table 2, serum cholesterol fractions (TC, LDL, HDL, and VLDL) were observed to be significantly elevated (*P* < 0.01) above normal control level in the infected control mice and infected mice treated with CQ (Table 1). However, significant decreases (*P* < 0.05) were observed in serum TC, LDL, HDL, and VLDL levels in infected mice treated with aqueous extract of *Ganoderma lucidum* (at 100, 250 and 500 mg/kg body weight) compared to infected untreated mice though these values were significantly higher compared to normal control level. Serum TC and LDL-C concentrations were not significantly (*P* > 0.05) changed in mice treated with 100 mg/kg and 250 mg/kg body weight of the extract. The extract at 100 mg/kg and 250 mg/kg body weight produced significantly lower reductions in serum TC and LDL-C compared to 500 mg/kg extract concentration.

As shown in Table 3, liver TC, LDL-C, HDL-C, and VLDL-C concentrations were significantly (*P* < 0.01) higher in infected control mice compared with normal control. The liver concentration of these cholesterol fractions were significantly (*P* < 0.05) reduced in *P. berghei* infection as a result of treatment with aqueous extract of *G. lucidum*. Liver TC, LDL, HDL and VLDL cholesterol were significantly (*P* < 0.05) higher in infected mice treated with 100, 250, and 500 mg/kg body weight *G. lucidum* aqueous extract compared to those administered CQ.

## 4. Discussion 

 Lipids have been observed to play important roles in pathological changes observed in disease conditions and are implicated in the production of immunity against diseases [23]. Serum lipids primarily bound to lipoproteins can be elevated by an increase in biosynthesis and/or by a decrease in their removal. Both of these processes appear to contribute to the hyperlipidemia that is often produced by some pathological changes. Although the source of increase in erythrocyte lipid component in malaria infection is not from lipid of the parasite there is no indication that the increase in the serum lipid is due to the lipid content of the parasite [24]. 

 The results obtained from the suppressive, curative, and prophylactic tests indicate that the aqueous, chloroform and ethanol extracts of *G. lucidum* possessed significant antiplasmodial activity as evident from the chemosuppression obtained during the four day early infection test. However, on established infection the plant extracts also exhibited significant activity as evident from the curative effect of the extracts. Agents with suppressive activity against *Plasmodium berghei* were known for antimalarial activity [21]. It is noteworthy that the antiplasmodial activity of the extract at all doses during early and established infections was comparable to that of the standard drug, CQ. There was a dose-dependent chemosuppression of parasitemia seen with the extract. In infected untreated mice, the parasite count increased daily until the death of the animals which was also observed in other studies [25, 26]. 

It is evident based on the findings from this study that aqueous extract of the fruiting bodies of *G. lucidum* represent potential antiplsmodial agent. The activity (antiplasmodial) may be attributed to terpenes, sterols, and flavonoids present in the extract as confirmed in this study. Antiplasmodial screening of plant substances has implicated terpenes, flavonoids and alkaloids [27–29]. These compounds could be acting singly or in synergy with one another to exert the antiplasmodial activity observed in this study. 

 Results obtained in the present study show significant increases in serum and liver total, LDL, VLDL, and HDL cholesterol in mice infected with *Plasmodium berghei*. This observation is consistent with reports from earlier studies by Onyeneke et al. [30] and Onongbu and Onyeneke [31] which showed increased serum lipoprotein fractions in malarial patients compared with apparently healthy control subjects. The observed increases in hepatic lipoproteins content are also in agreement with the report of Lombard et al. [8]. According to Lombard et al. [8] increase in hepatic lipid in malarial subjects is consistent with the degree of parasitemia. Again, observations from the present study support this assertion as the level of parasitemia correlates with hepatic lipoprotein composition. 

 As observed from this study, administration of aqueous extract of *G. lucidum* downregulates serum and liver lipoprotein content. The observed effect of the extract was to be negatively correlated with parasitemia level. The hypocholesterolemic property of *G. lucidum* has been established in our earlier study [19]. 

 Malaria disturbs liver function though there is no evidence of hepatic insufficiency. In malarial infection, the liver is commonly enlarged and congested with parasitized red cells in sinusoids and centrilobular veins and swollen parenchymatous and kupffer cells [32]. Lipid metabolism is therefore deranged and the resulting lipoprotein particles synthesized by the liver undergo drastic changes in composition. Maurois et al. [33] have demonstrated that increased lipolysis induced by threshold of parasitemia promotes VLDL synthesis and their metabolism into LDL. Thus, with increase in parasitemia there was a progressive increase in LDL fraction and with the resulting incorporation of cholesterol particles, the overall LDL cholesterol was found to be increased. There is also the possibility of a reduction in the breakdown of LDL by the infective liver tissue thereby leading to an increase in these particles. It is also likely that the elevation in LDL cholesterol may be due to decrease uptake by the infected erythrocytes as a result of increased levels of parasitemia. 

 In conclusion, results obtained from this study show that aqueous extract of *G. lucidum* reduces serum and liver lipoprotein cholesterol with a corresponding decrease in parasitemia levels in both tissues. Therefore, it could thus be inferred that the antimalarial property of the extract could possibly be mediated by mechanisms involving reduction in liver lipids. 

## Figures and Tables

**Table 1:** Suppressive effect of aqueous extract of *Ganoderma lucidum* on parasitemia in mice infected with *Plasmodium berghei*.

Treatment groups		Parasite count	% suppression
Controls	Normal (P-CTR)	—	—
	Infected (N-CTR)	31.10 ± 1.20	—
Aqueous extract	AQ_100_	14.20 ± 0.60	54.20 ± 3.70^a^
	AQ_250_	17.40 ± 1.20	43.82 ± 6.03^a,b^
	AQ_500_	19.40 ± 0.90	37.42 ± 5.31^b,c^
Reference drug	CQ	2.25 ± 0.85	93.65 ± 0.47^c^

Results are means ± SEM of eight independent determinations. Values in the same row carrying different superscripts are significant (*P* < 0.05) from each other.

Note. AQ_100_: infected mice treated with 100 mg/kg aqueous extract; AQ_250_: infected mice treated with 250 mg/kg aqueous extract; AQ_500_: infected mice treated with 500 mg/kg aqueous extract; CQ: infected mice treated with chloroquine.

**Table 2:** Serum TC, LDL-, HDL-, and VLDL-cholesterol levels in mice *Plasmodium berghei* infected mice treated with aqueous extract of *Ganoderma lucidum*.

Treatment groups		TC	LDL	HDL	VLDL
Controls	Normal	8.75 ± 0.86^a^	5.29 ± 0.57^a^	1.46 ± 0.01^a^	2.01 ± 0.01^a^
	Infected	68.43 ± 5.33^b^	36.56 ± 5.64^b^	16.14 ± 0.01^b^	15.74 ± 0.01^b^
Aqueous extract	AQ_100_	33.76 ± 2.27^c^	21.77 ± 1.47^c^	3.88 ± 0.01^c^	8.10 ± 0.17^c^
	AQ_250_	39.97 ± 1.29^c^	25.58 ± 0.83^c^	4.40 ± 0.01^d^	9.99 ± 0.06^d^
	AQ_500_	63.87 ± 5.03^b^	40.87 ± 3.22^b^	8.33 ± 0.01^e^	14.69 ± 0.01^e^
Reference drug	CQ	66.17 ± 4.50^b^	39.86 ± 2.40^b^	10.43 ± 0.01^f^	15.88 ± 0.02^b^

Results are means ± SEM of eight independent determinations. Values in the same row carrying different superscripts are significant (*P* < 0.05) from each other.

Note. P-CTR: positive control mice; N-CTR: negative control mice; AQ_100_: infected group treated with 100 mg/kg *G. lucidum*; AQ_250_: infected group treated with 250 mg/kg *G. lucidum*; AQ_500_: infected mice treated with 500 mg/kg *G. lucidum*; CQ: infected mice treated with chloroquine.

**Table 3:** Liver TC, LDL-, HDL-, and VLDL-cholesterol levels in *Plasmodium berghei*-infected mice treated with aqueous extract of *Ganoderma lucidum*.

Treatment groups		TC	LDL	HDL	VLDL
Controls	Normal	66.03 ± 6.97^a^	37.51 ± 3.82^a^	12.67 ± 0.01^a^	15.85 ± 0.06^a^
	Infected	171.78 ± 8.70^b^	99.25 ± 8.46^b^	31.31 ± 0.01^b^	41.22 ± 0.02^b^
Aqueous extract	AQ_100_	146.42 ± 4.34^c^	93.70 ± 2.78^b^	19.29 ± 0.25^c^	33.68 ± 0.02^c^
	AQ_250_	150.94 ± 9.54^b,c^	96.60 ± 6.10^b^	16.60 ± 0.11^d^	37.74 ± 0.02^d^
	AQ_500_	172.00 ± 8.35^b^	110.08 ± 5.34^b^	22.36 ± 0.01^e^	39.56 ± 0.06^e^
Reference drug	CQ	111.56 ± 4.99^d^	71.40 ± 3.19^c^	13.39 ± 0.01^f^	26.78 ± 0.02^f^

Results are means ± SEM of eight independent determinations. Values in the same row carrying different superscripts are significant (*P* < 0.05) from each other.

Note. P-CTR: positive control mice; N-CTR: negative control mice; AQ_100_: infected group treated with 100 mg/kg *G. lucidum*; AQ_250_: infected group treated with 250 mg/kg *G. lucidum*; AQ_500_: infected mice treated with 500 mg/kg *G. lucidum*; CQ: infected mice treated with chloroquine.
